# Blood group profile of Türkiye with regional and demographic differences: a 23-year retrospective study

**DOI:** 10.55730/1300-0144.6049

**Published:** 2025-07-09

**Authors:** Levent SAĞDUR, Aziz KARACA, Mustafa Nuri GÜNÇIKAN, Mustafa YILMAZ, Kerem KINIK

**Affiliations:** 1 Division of Medical Management Directorate, Department of General Directorate of Blood Services, Turkish Red Crescent, Ankara, Turkiye; 2 Department of Physiology, Faculty of Medicine, Recep Tayyip Erdoğan University, Rize, Turkiye; 3 Department of Emergency Aid and Disaster Management, Faculty of Medicine, Health Sciences University, İstanbul, Turkiye

**Keywords:** Blood stock management, donor demographic analysis, Turkish blood donors, Turkish blood groups

## Abstract

**Background/aim:**

This study aimed to comprehensively analyze the distribution of ABO and Rh blood groups, as well as demographic characteristics and regional variations, using data from 9.5 million blood donors across Türkiye.

**Materials and methods:**

In this multicenter, retrospective, cross-sectional study, data were collected from 9,587,592 healthy, voluntary donors who donated to the Turkish Red Crescent between 1998 and 2021. Donors met the criteria of being 18–70 years old, weighing over 50 kg, with hemoglobin levels above 13.5 g/dL for males and 12.5 g/dL for females. The data were evaluated based on age, sex, education level, and geographic region.

**Results:**

The ABO blood group distribution was determined as follows: A (42.1%), O (34.0%), B (16.4%), and AB (7.5%). When analyzed together with the Rh factor, A Rh (+) was the most common (37.1%), while AB Rh (−) was the least common (0.9%). Regional analyses showed that A Rh (+) was highest in the Black Sea Region, whereas the Aegean Region had a notably high AB Rh (+) rate. The Marmara Region had the highest total number of donors, while Eastern Anatolia had the highest donation rate relative to its population. Demographically, 82.0% of donors were male; the 18–34 age group represented the most active segment (50.5%), and high school graduates had the highest donation rate (31.2%).

**Conclusion:**

This study provides a detailed profile of Türkiye’s blood donors, filling a gap in national blood group data. The findings support optimizing transfusion services, improving blood supply management, and refining donor recruitment strategies. Regional and demographic disparities highlight the need for targeted awareness campaigns. Addressing sex imbalances and increasing participation from underrepresented groups can enhance sustainability. Additionally, these findings contribute valuable data for clinical applications, epidemiological studies, and future healthcare policy development.

## 1. Introduction

The Turkish Red Crescent (TRC) plays a crucial role in blood banking. Since the establishment of the first blood bank in the 1950s, blood donation and storage processes have become systematic nationwide. The integration of modern technologies in the 2000s improved blood donation processes, leading to significant advancements in blood component separation. Over the years, standards and regulations have been implemented to ensure blood safety. In this context, the “Safe Blood Project” studies were initiated by the TRC and the Ministry of Health of the Republic of Türkiye on June 1, 2005 [1]. The TRC’s efforts have been vital in raising public awareness about blood donation and ensuring a sufficient supply in emergencies.

The TRC collects blood from healthy, voluntary, and nonremunerated donors, supported by educational sessions, public service announcements, and promotional activities. On average, regular, first-time, and occasional donors constitute 53%, 26%, and 21% of donors, respectively. While the TRC supplies 90% of the blood demand of hospitals, the remaining portion is met by 14 Temporary Regional Blood Centers.

While blood group distributions have been reported in various regions of Türkiye, a nationwide study with a large sample size encompassing all regions has not been conducted. This study aimed to determine the ABO and Rh blood group distribution across the country, making it the most comprehensive research performed to date on this topic. By including demographic data such as age, sex, and educational status, this study not only fills the gaps left by previous research but also contributes to transfusion medicine practices and the country’s blood group database [2–10].

## 2. Materials and methods

### 2.1. Data collection

This retrospective, cross-sectional study includes 9,587,592 blood donors who donated to the TRC between 1998 and 2021. As part of the Safe Blood Project launched in 2005, systematic data recording primarily began in 2005, although TRC had conducted blood donation activities before this date. Therefore, in this study, data collected irregularly between 1998 and 2005 were generally referred to as 2005 data. Only one donation per donor was included to avoid bias. The data analyses categorized the regional blood group distribution, education level, and sex based on Türkiye’s seven geographical regions. Additionally, to ensure a balanced distribution across age groups, two categories were created: 18–34 years and ≥35 years.

### 2.2. Ethical approval

Ethical approval was obtained from the TRC Research Ethics Committee (ethical approval number: 2023/06, date: November 7, 2023).

### 2.3. Data sources and techniques

Data were obtained from the TRC database. Immunohematology techniques used for blood grouping during the study years are shown in Table S1.

### 2.4. Statistical analysis

Categorical variables were analyzed as frequencies and percentages. All analyses were performed using OpenEpi (v 3.0.1) to summarize the demographic characteristics and blood group distribution of the donors.

## 3. Results

### 3.1. Regional distribution of blood donors

Since 2005, the number of blood donors has continuously increased (Figure). Between 1998 and 2021, the highest number of donors was in the Marmara Region [2,791,405 (29.1%)], while the lowest number of donors was in the Eastern Anatolia Region [745,387 (7.8%)] (Table 1). However, in 2021, the Eastern Anatolia Region recorded the highest donation rate relative to its population [5.1% (157,654 of 3,096,985)], while Southeastern Anatolia had the lowest [3.3% (200,936 of 6,046,425)] (Table 2).

### 3.2. Sex and age distribution

First, it should be noted that the numerical differences between the tables are due to whether or not the donors marked the relevant field on the form. Comparing the TRC’s blood donors aged 18–70 (between 1998 and 2021) with Türkiye’s total population in the same age range, men donated significantly more than women, and the 18–34 age group donated more than those aged ≥35. Regionally, the Southeastern Anatolia Region had the highest proportion of male donors [657,929 (85.0%)], while the Black Sea Region has the highest proportion of female donors [203,251 (21.1%)]. The 18–34 age group had the highest donation rate in Eastern Anatolia [478,532 (64.2%)] and the lowest was in Marmara [1,289,213 (46.2%)] (Table 1).

### 3.3. Education level and donation rates

Between 1998 and 2021, the TRC blood donors were categorized by education level according to the International Standard Classification of Education (ISCED)^1^. High school graduates had the highest donation rate [2,908,578 (31.2%)], while doctoral degree holders have the lowest [43,735 (0.5%)] (Table 3). When analyzing the results by region, it was found that the highest donation rates for literate, primary school, and middle school were in the Southeastern Anatolia Region [17,169 (2.3%); 169,931 (22.5%); 116,660 (15.4%); respectively], while high school had the highest donation rate in the Mediterranean Region [440,175 (33.1%)]. The highest donation rates for associate’s degree and bachelor’s degree individuals were in the Black Sea Region [120,928 (12.9%); 260,358 (27.7%)]. The highest proportion of donors with a master’s degree was in the Marmara Region [77,778 (2.8%)], and Central Anatolia Region [15,139 (1.1%)] had the highest proportion of donors with a doctorate degree.

### 3.4. Blood type distribution and regional variations

Although the distribution of blood types showed regional variations, A Rh (+) [3,562,627 (37.1%)] was the dominant group nationwide, followed by O Rh (+) [2,851,802 (29.7%)]. The least common blood types were AB Rh (−) [87,685 (0.9%)] and B Rh (−) [184,598 (1.9%)]. In terms of regional differences according to the proportion of the regional total population, the Black Sea Region had the highest proportion of A Rh (+) [366,352 (38.0%)], while the Aegean Region had the highest percentage of AB Rh (+) [105,784 (7.0%)]. The lowest O Rh (+) rate was found in the Central Anatolia Region [407,436 (28.7%)], whereas the highest O Rh (+) percentage was in Southeastern Anatolia [241,303 (31.2%)]. Additionally, the highest proportion of the O Rh (−) blood group was observed in the Black Sea and Eastern Anatolia Regions [47,324 (4.9%) and 30,141 (4.9%), respectively], while the Aegean Region had the lowest proportion of O Rh (−) [57,609 (3.8%)] (Table 4).

## 4. Discussion

The focus of this study was the comprehensive analysis of ABO and Rh blood group distribution data in Türkiye, utilizing a larger sample size and broader geographical coverage than previous studies [2–10]. By categorizing data based on demographics such as age, sex, and education level, this research addresses gaps from earlier studies. The detailed regional breakdown enhances our understanding of blood donor profiles, informing targeted strategies for donor recruitment and transfusion practices.

Since 2005, the TRC’s safe blood program has effectively met the country’s blood needs [1], with an increasing number of blood donors, similar to trends in other countries [11]. Blood donations decreased in 2020 due to the COVID-19 pandemic. However, donation rates have started to rise again since 2021, following effective promotional activities organized by the TRC.

The Marmara Region, with the largest population, has the most blood donations, while the lowest occur in Eastern Anatolia. A similar pattern is seen in other large countries [12,13]. Despite Marmara’s high numbers, Eastern Anatolia has the highest per capita donation rate, while Southeastern Anatolia and Marmara have the lowest. This suggests that donation rates are influenced by promotional activities and awareness programs, pointing to the need for targeted strategies in low-rate regions.

Analysis of the national dataset shows a notable disparity in the sex distribution of blood donors in Türkiye, with a significantly higher proportion of male donors compared to female donors. Multiple studies conducted in Türkiye corroborate this finding. For instance, one study reported that 89.1% of blood donors were male [14], while another study focusing on first-time donors found that 76.8% were male [15]. Similarly, a study in İstanbul indicated that 92.42% of blood donors were male [16], and another study in the same city reported 93.27% male donors [17]. This trend of male predominance appears consistent across various regions and studies within Türkiye. Again, like in many other countries^2^,^3^,^4^, Türkiye has a higher proportion of male blood donors compared to females. In contrast, some international studies show different patterns. For example, a study in Brazil found that 72.8% of participants were female, although men donated more frequently [18]. This suggests that while the willingness to participate about blood donation might differ by sex in some contexts, the actual act of donating blood in Türkiye is more prevalent among males. This highlights potential cultural differences in sex roles and attitudes towards blood donation. Additionally, this may be attributed to more restrictive criteria for female donors, such as those related to anemia, pregnancy, and breastfeeding. However, this disparity is less pronounced in other countries (for example, male/female ratios of 56.4:43.6% in Canada^2^, 54.9:45.1% in Ireland^3^, 53.3:46.7% in Hong Kong^4^, and 45.9:54.1% in the USA^5^). Clearly, Türkiye needs more effective campaigns to encourage greater female participation in blood donation.

Demographic factors, especially education and age, can influence willingness to donate blood [19]. In our study the donation rate for the 18–34 age was nearly the same as for the ≥35 age. Numerous studies conducted in Türkiye support this finding [2–10] that contrasts with trends in other countries^3^,^4^,^5^. The high donation rate among young people, especially in Eastern and Southeastern Anatolia, is promising for future donor recruitment. A study analyzing blood donors in İstanbul found that 69.28% were in the age group of 25–44 years [16]. A study examining first-time donors in Türkiye found that participants were between 18 and 65 years old, with the 35–44 age group showing the highest intention to donate again [15]. This aligns with another Turkish study where the mean age of donors was 27 years, and the majority (75.47%) were between 18 and 30 years old [20]. While international data shows variations, such as a study in Atlanta where the highest percentage of donors were in the 40–49 age group [21], the trend in Türkiye appears to be a younger donor demographic. The age distribution of donors also varies internationally. While Türkiye sees a significant contribution from younger to middle-aged adults, high-income countries like the US have observed an increase in older donors^5^, and in low- and middle-income countries, more young people donate proportionally^6^. These differences could be linked to factors such as targeted recruitment strategies, the overall age structure of the population, and the impact of events like school-based blood drives^5^. Socioeconomic factors influencing blood donation also show both similarities and differences across countries. While employment and income have been observed to be positively associated with donation in the United States and Jordan [22], an interesting trend in Türkiye indicates that unemployed donors have a higher intention to donate compared to employed donors [15].

Similar to a previous study [23], our study found that most donors were secondary school graduates, while fewer were primary school graduates. The education level of blood donors in Türkiye suggests a significant representation from individuals with high school and university education. A study in Türkiye on first-time donors found that 41.3% had a high school diploma, and 28.8% were attending university [15]. In a survey study on blood donation attitudes, among those who had previously donated blood, the proportion of high school and university graduates was 40.2%, while the proportion of those who had not graduated from school was 37.5% [24]. This trend is also observed in other regions; for example, a study in Jordan found a high proportion of donors with high school and university education [22]. Similarly, research in Brazil showed that a large percentage of participants had completed high school or had university education [18]. These findings suggest a potential correlation between higher levels of education and the propensity to donate blood. This highlights the need to increase awareness about blood donation among those with lower levels of education.

Consequently, the similarities and differences in blood donation behavior based on demographic factors such as age, sex, and education level likely stem from the complex interplay of cultural norms, socioeconomic conditions, healthcare policies, individual motivations, and barriers related to donation. Altruism and a sense of social responsibility are frequently cited as key motivations for blood donation across various cultures. However, barriers such as fear of needles, lack of time, and concerns about medical eligibility also play a significant role in deterring potential donors. Cultural beliefs and sex roles can further influence these motivations and barriers, as seen in the fear of anemia among women in Brazil [18] or the historical underrepresentation of minority donors in the US due to systemic racism and mistrust^7^. Understanding these nuances is crucial for developing effective strategies to promote blood donation among specific demographic groups in Türkiye and worldwide.

In our study, the ABO blood group distribution was ranked as follows: A (42.1%) > O (34.0%) > B (16.4%) > AB (7.5%). When compared with other previous studies conducted in Türkiye, a partial similarity in the distribution rates is observed (Table S2). The national distribution of ABO and Rh blood groups among donors in Türkiye is consistent with general population data. Studies conducted in different regions of Türkiye, such as Istanbul and Eastern Türkiye, show similar frequencies for blood groups A, O, B, and AB, as well as Rh (+) rates. Typically, blood group A is the most frequent, followed by O, then B, and AB being the least common. Rh (+) is also high [16,25]. Other countries’ blood group distribution data, based on the most recent available data at the time of writing this article, are presented in Table S3. Switzerland had the highest percentage of group A (45.2%), while Ghana had the lowest group A percentage (20.6%). India had the highest percentage of group B (32.3%), whereas Mexico had the lowest group B percentage (8.9%). Japan had the highest percentage of group AB (9.9%), while Mexico had the lowest group AB percentage (1.8%). Furthermore, Mexico had the highest percentage of group O (62.0%), while Japan had the lowest group O percentage (29.3%). These variations worldwide are significant in terms of showing ethnic/racial differences. In conclusion, when examining the data, it is notable that group A is more prevalent in Türkiye, while group O is more common globally.

Our study also shows regional differences in blood group distribution. The prevalence of O and B blood groups in the Eastern and Southeastern provinces of Türkiye may be explained by the population of Arabic and Kurdish descent who have a dominance of blood groups O and B [26–28]. Since the donors are not required to indicate their ethnic origin before or during donation process, the correct ethnic distribution in the regions is not known. Similar differences have been observed in other countries [12,13,28–31]. On the other hand, although studies conducted in Türkiye do not comprehensively show the nationwide geographic distribution of blood donors, one study provides insights into organ donation rates across the country. According to this study, the highest donation rates were observed in the Aegean and Marmara regions, while the lowest rates were recorded in the Eastern and Southeastern Anatolia regions [32]. Similar regional disparities are likely to be seen in blood donation rates as well, as factors such as awareness of donation, accessibility of donation centers, and cultural norms vary across different regions of the country. If the national dataset includes geographic information, a detailed analysis of these regional differences would provide valuable insights for targeted donor recruitment strategies.

### 4.1. Limitations and strengths

Our study was restricted to individuals between the ages of 18 and 70 who donated blood to the TRC. Furthermore, because of the disproportionate representation of male donors, the findings may not be applicable to both sexes. On the other hand, the study has significant strengths, such as a substantial sample size and the comprehensive analysis of data collected from a wide geographical range.

## 5. Conclusion

TRC effectively manages blood supply through meticulous analysis of parameters such as age, sex, education level, and blood group distribution among blood donors, who provide approximately 90% of Türkiye’s blood supply. We believe that the findings from this study are significant for the planning and implementation of activities carried out by TRC. Furthermore, considering that the distribution of blood groups in Türkiye has not been previously documented on such a scale and with such geographical diversity, this study provides crucial information that can potentially be used in clinical research. Furthermore, this study shows that the ratio of ABO and Rh blood groups were relatively close to each other in different geographical regions, contributing to the understanding of Türkiye’s blood group distribution.

## Supplementary materials

**Table S1:** Test methods and kits used in immunohematologic blood group typing.

Methods	Device	Kit	Year
Microplate (Forward)	Beckman Coulter PK7400 (Japan)	Diagast (France)	Since 2021
Microplate (Forward, Weak D, Antibody Screening)	Neo (Fully Automatic, Germany)	Immucord (Germany)	2016–2020
Gel Centrifugation (Forward-Reverse, Weak D, Rh Subgroup, Antibody Screening)	Grifols / Erytra (Fully Automatic, Spain)	Grifols (Spain)	Since 2015
Gel Centrifugation (Forward-Reverse, Weak D, Rh Subgroup)	Gel Station (Fully Automatic) Swing-Saxo-Banjo (Semi-Automatic) Techno (Fully Automatic, Switzerland)	DiaMed, BioRad (Switzerland)	2008–2015
Column Agglutination Method (Forward-Reverse, Rh Subgroup) (With Weak D Manual Card)	Ortho Diagnostics Auto Vue Innova Device (Fully Automatic, USA)	Ortho Bio Vue (USA)	2006–2008
Tube Method (Weak D)	Manual Method	DiaMed Coombs Serum (Switzerland), Tulip Anti-D (India)	2005–2006
Tumerblood Plate (Forward-Reverse)	Manual Method	Antiserum Tulip (India), A1 and B cells for reverse grouping are prepared in the laboratory.	1998–2006

**Table S2:** ABO and Rh blood group distribution throughout Türkiye.

Study	Year	City	n	A Rh (+) %	A Rh (−) %	B Rh (+) %	B Rh (−) %	AB Rh (+) %	AB Rh (−) %	O Rh (+) %	O Rh (−) %
This study	2025	All of Türkiye	9,587,592	37.1	5.0	14.4	1.9	6.6	0.9	29.7	4.2
Babacan [ 1]	1968	All of Türkiye	20,989	39.0	4.3	14.5	1.8	7.0	1.0	29.0	3.4
Sanli [ 2]	2025	İstanbul	507,959	44.2		15.2		7.1		33.6	
Ergün & Yardımcı [ 3]	1993	Ankara	288,469	39.9	4.7	14.1	1.4	6.3	1.4	27.9	4.3
Tapan [ 4]	2019	İzmir	232,364	37.8	5.0	14.0	2.0	6.8	0.9	29.3	4.2
Balcı et al. [ 5]	2010	Denizli	64,840	38.8	3.8	14.8	2.0	6.5	0.9	29.8	3.5
Altuner Torun et al. [ 6]	2012	Kayseri	86,797	44.0		16.2		6.5		33.3	
Aktaş & Ünlü [ 7]	2021	Sivas	50,441	38.4	5.5	14.2	2.0	6.8	0.9	27.8	4.1
Kader et al. [ 8]	2014	Yozgat	5257	38.5	5.8	14.0	1.9	7.3	0.8	28.3	3.4
Gündem & Ataş [ 9]	2019	Konya	30,291	37.0	4.9	16.1	1.8	7.3	0.9	28.4	3.6
Coşkun [ 10]	1990	Gaziantep	33,317	36.3	3.7	16.4	1.7	6.2	0.6	32.0	3.1
Arac et al. [ 11]	2019	Diyarbakır	127,091	35.3	5.6	16.4	2.3	7.1	1.0	29.7	4.0
Zerin et al. [ 12]/	2004	Şanlıurfa	28,944	36.4		21.3		7.7		34.7	
Alpdemir et al. [ 13]	2014	Balıkesir	128,862	38.0	4.7	16.4	1.8	7.4	1.0	27.1	3.6
Koçak et al. [ 14]	2017	Erzurum	69,686	39.5	6.5	12.7	2.0	6.5	1.0	27.1	4.4
Kuku et al. [ 15]	2004	Malatya	65,277	36.9	4.3	13.5	1.5	5.8	36.9	4.3	13.5
Akın & Nostbil [ 16]	2003	Van	6982	35.5	4.5	15.8	1.3	13.5	1.2	24.8	3.5
Özkasap et al. [ 17]	2013	Rize	38,329	37.0	7.0	8.0	1.3	2.2	0.4	36.6	7.5
İnci & Karataş [ 18]	2020	Karabük	8575	40.4	4.3	13.9	2.0	13.3	1.0	22.2	2.9
Yıldız [ 19]	2016	Adana	136,038	38.9		17.0		6.9		37.1	
Koçtekin B. [ 20]	2020	Antalya	10,867	39.0	4.0	14.3	1.3	5.7	0.8	31.4	3.5

**Table S3:** Blood group distribution of countries.

Countries	A Rh (+) (%)	Arh (−) (%)	B Rh (+) (%)	B Rh (−) (%)	AB Rh (+) (%)	AB Rh (−) (%)	O Rh (+) (%)	O Rh (−) (%)
**T** **ü** **rkiye (This study)**	37.1	5.0	14.4	1.9	6.6	0.9	29.7	4.2
**U.S.A. (Total) [ 1]**	32.0	6.0	11.0	2.0	4.0	1.0	39.0	7.0
**U.S.A. (Caucasian) [ 2]**	33.0	7.0	9.0	3.0	3.0	1.0	37.0	8.0
**U.S.A. (African-American) [ 2]**	24.0	2.0	18.0	1.0	4.0	0.3	47.0	4.0
**U.S.A. (Asian) [ 2]**	27.0	0.5	25.0	0.4	7.0	0.1	39.0	1.0
**U.S.A. (Latino-American) [ 2]**	29.0	2.0	9.0	1.0	2.0	0.2	53.0	4.0
**Mexico [ 3]**	26.1	1.4	8.5	0.4	1.7	0.1	59.3	2.7
**Venezuela [ 4]**	30.1		10.4		2.2		57.3	
**Canada [ 5]**	36.0	6.0	7.6	1.4	2.5	0.5	39.0	7.0
**United Kingdom [ 6]**	30.0	8.0	8.0	2.0	2.0	1.0	35.0	13.0
**İ** **reland [ 7]**	22.8	6.0	8.7	2.6	1.8	0.6	43.1	14.4
**Netherlands [ 8]**	30.3	8.0	8.1	1.7	3.5	0.8	35.8	11.8
**Belgium [ 9]**	37.0	7.0	9.0	1.0	3.0	1.0	36.0	7.0
**Switzerland [ 10]**	38.3	6.9	8.4	1.4	3.5	0.6	34.8	6.1
**Australia [ 11]**	32.0	6.0	12.0	2.0	4.0	1.0	38.0	7.0
**Saudi Arabia [ 12]**	26.9		20.4		5.2		47.5	
**China [ 13]**	28.7		28.2		8.9		34.2	
**Japan [ 14]**	38.7		22.1		9.9		29.3	
**Bangladesh [ 15]**	26.6		23.2		9.6		40.6	
**India [ 16]**	22.9		32.3		7.7		37.1	
**Iran [ 17]**	30.2		27.7		8.3		33.8	
**Northern Region of Iraq [ 18]**	29.8	2.7	21.1	1.7	7.1	0.5	33.2	3.8
**Nigeria [ 19]**	22.8		20.6		3.7		52.9	
**Mauritania [ 20]**	26.6	1.7	17.5	1.1	3.9	0.2	46.2	2.8
**Ethiopia [ 21]**	26.0	3.0	20.0	2.2	7.0	0.7	39.0	3.0
**Ghana [ 22]**	18.9	1.7	24.4	1.3	3.1	0.1	46.3	4.4

References1

BabacanM

Distribution of ABO Rh Blood Groups In Türkiye and Their Distribution according to Geographical Regions (In Turkish)
The Eurasian Journal of Medicine
1968
1
27
36
2

SanliK

Changes in Blood Groups of Patients Cared for in a Third Level Hospital in Istanbul Over the Years
Clin Lab
2025
71
3
10.7754/Clin.Lab.2024.241030
400665453

ErgünA
YardımcıS

Distribution of ABO Blood Groups and Rh Factor in Türkiye (In Turkish)
Ankara Tıp Mecmuası
1993
46
527
533
4

TapanYU

Investigation of the distribution of abo and kell blood groups and rh subgroups of patients and donors applied to Dokuz Eylul University Medical Faculty Hospital (In Turkish)
Dokuz Eylul University Faculty of Medicine
İzmir
2019
5

BalcıYI
ÖvetG
ÇövütİE
GoncuF
YilmazM

ABO and Rh Blood Groups Frequency in Denizli Province
International Journal of Hematology and Oncology
2010
20
103
105
6

Altuner TorunY
KaynarLG
KarakukcuC
YayM
KurnazF


ABO and Rh Blood Group Distribution in Kayseri Province, Turkey
Turk J Haematol
2012
29
 1
97
98
10.5505/tjh.2012.26918
24744637
PMC39867827

AktaşA
ÜnlüG

Evaluation of the Distribution of ABO and Rh Blood Groups in Sivas Province
Cumhuriyet Medical Journal
2021
43
 1
55
61
10.7197/cmj.887707
8

KaderÇ
YolcuS
DoğanB
PınarbaşlıM
İlanbeyB


ABO and Rh Blood Groups Distribution in Yozgat City, Turkey
Journal of Clinical and Experimental Investigations
2014
5
 2
169
172
10.5799/ahinjs.01.2014.02.0384
9

GündemNS
AtaşE

Distribution of ABO and Rh Blood Groups among Patients Admitted to a Gynaecology, Obstetrics and Children Hospital in Konya, Turkey
Journal of Clinical & Diagnostic Research
2019
13
 3
1
4
10

CoşkunY

The Distribution of “ABO” and “Rh” Blood Group in Gaziantep Region (In Turkish)
Gaziantep Üniversitesı Tıp Fakültesi Dergisi
1990
1
13
15
11

AracE
SolmazI
SamanciS

ABO and Rh blood groups frequency in men, women and neonates in Diyarbakir province
Annals of Medical Research
2019
26
 12
2876
2880
10.5455/annalsmedres.2019.09.538
12

ZerinM
KarakılçıkAZ
NazlıgülY

Frequency of ABO and Rh blood groups in Sanliurfa region (In Turkish)
Harran Üniversitesi Tıp Fakültesi Dergisi
2004
1
 3
15
17
13

AlpdemirM
AlpdemirMF
KocaözS
ErmişT
AtlıA

ABO and Rh Blood Group Dıstrıbutıon In Balıkesır Provınce, Turkey (In Turkish)
Balıkesir Sağlık Bilimleri Dergisi
2014
3
 2
69
73
14

KocakAO
OmerogluM
KatipogluB
AkbasI
CanNO

Blood Group Analysis of Patients Applying to Erzurum Ataturk University Hospital
Research and Reports of Medical Science
2017
1
 1
1
4
15

Kukuİ
KayaE
ErkurtMA
DikilitaşM
YıldızR


ABO and Rh Blood Group Distribution in Malatya Region (In Turkish)
İnönü Üniversitesi Tıp Fakültesi Dergisi
2004
11
 4
213
215
16

AkınG
DostbilN

Research Of The Blood Groups In Turkey (In Turkish)
Yuziincil Yil Universitesi Fen Bilimleri Dergisi
2003
8
 1
28
36
17

ÖzkasapS
DereciS
ŞahinK
DilekAR
KalyoncuoğluE


Analysis of ABO and Rh blood groups distribution in East Karadeniz region of Turkey (In Turkiye)
Dicle Medical Journal
2013
40
 1
100
104
10.5798/diclemedj.0921.2013.01.0232
18

İnciF
KarataşF

Distribution of ABO and Rhesus Blood Groups in Cancer Patients (In Turkish)
Uludağ Üniversitesi Tıp Fakültesi Dergisi
2020
46
 3
379
384
10.32708/uutfd.812217
19

YıldızŞM

Distribution of ABO and Rh blood group systems in Cukurova region (In Turkish)
Cukurova Medical Journal
2016
41
 4
658
663
20

KoçtekinB

Investigation of ABO and Rhesus (Rh) blood group distribution in donors applying to Antalya Education and Research Hospital Transfusion Center (In Turkish). (In Turkish)
Mersin Üniversitesi Saglık Bilimleri Dergisi
2020
13
 3
395
403


References1
America’s Blood Center
2024
U.S. Blood Donation Statistics and Public Messaging Guide [online]
Website https://americasblood.org/wp-content/uploads/2022/05/Whitepaper-National-Stats_5.22.pdf
accessed 10 July 2025
2
American Red Cross
2025
Facts About Blood and Blood Types [online]
Website https://www.redcrossblood.org/donate-blood/blood-types.html
accessed 10 July 2025
3

Canizalez-RomanA
Campos-RomeroA
Castro-SanchezJA
Lopez-MartinezMA
Andrade-MunozFJ


Blood Groups Distribution and Gene Diversity of the ABO and Rh (D) Loci in the Mexican Population
Biomed Research International
2018
2018
1925619
10.1155/2018/1925619
29850485
PMC59375184

TeodoroV
MilagrosC
LeoxinéP
AurimarD
AnnyP


Distribución de grupos sanguíneos ABO y Rh en candidatos a donantes de el Tocuyo, Venezuela (In Español)
Revista Venezolana De Salud Pública
2019
7
 2
9
16
5
Canadian Blood Services
2025
How Many Different Blood Types are There? [online]
Website https://www.blood.ca/en/blood/donating-blood/what-my-blood-type
accessed 10 July 2025
6
NHS Blood and Transplant
2025
Blood types [online]
Website https://www.blood.co.uk/why-give-blood/blood-types/
accessed 10 July 2025
7
Irish Blood Transfusion Service
2023
Annual Report [online]
Website https://www.giveblood.ie/media/publications/annual_reports/ibts-ar-2021-final-web.pdf
accessed 10 July 2025
8
Stichting Sanquin Bloedvoorziening
2023
Jaarverslag (in Dutch) [online]
Website https://www.sanquin.org/about-sanquin/publications/annual-reports
accessed 10 July 2025
9
Croix Rouge de Belgique
2025
Breakdown of the Blood Groups [online]
Website https://www.donneurdesang.be/en/find-out-more-about-blood/blood-groups
accessed 10 July 2025
10

VolkenT
CrawfordRJ
AmarS
MosimannE
TschaggelarA


Blood Group Distribution in Switzerland - a Historical Comparison
Transfusion Medicine and Hemotherapy
2017
44
 4
210
216
10.1159/000479191
28924425
PMC559794611
Australian Red Cross
2025
Blood types [online]
Website https://www.lifeblood.com.au/blood/learn-about-blood/blood-types
accessed 10 July 2025
12

KabrahSM
FlembanAF
KhogeerAA
BawazirWM

Reviewing Publication Discussing the Frequency of ABO and Rhesus-D Blood Groups in Saudi Arabia
Journal of Research in Medical and Dental Science
2021
9
 10
29
37
13

SunY
WangL
NiuJ
MaT
XingL


Distribution characteristics of ABO blood groups in China
Heliyon
2022
8
 9
e10568
10.1016/j.heliyon.2022.e10568
36119853
PMC947901914

FujitaY
TanimuraM
TanakaK

The distribution of the ABO blood groups in Japan
Jinrui Idengaku Zasshi
1978
23
 2
63
109
10.1007/BF02001790
691841
15

DewanG

Comparative frequency and allelic distribution of ABO and Rh (D) blood groups of major tribal communities of southern Bangladesh with general population and their determinants
Egyptian Journal of Medical Human Genetics
2015
16
 2
141
147
10.1016/j.ejmhg.2015.01.002
16

AgrawalA
TiwariAK
MehtaN
BhattacharyaP
WankhedeR


ABO and Rh (D) group distribution and gene frequency; the first multicentric study in India
Asian Journal Transfus Science
2014
8
 2
121
125
10.4103/0973-6247.137452
PMC41400552516135317

AndalibiM
DehnaviZ
AfshariA
TayefiM
EsmaeiliH


Prevalence of ABO and Rh blood groups and their association with demographic and anthropometric factors in an Iranian population: Mashad study
East Mediterr Health Journal
2020
26
 8
916
922
10.26719/emhj.20.048
3289688618

GettaHA
AminSS
KhoshnawN
MuhammadBA

Distribution of red cell antigens according to ABO, Rh and other rare blood group systems in Kurdish ethnicity
Iraq Joural of Hematology
2016
5
 1
55
80
19

AnifowosheAT
OwolodunOA
AkinseyeKM
IyiolaOA
OyeyemiBF

Gene frequencies of ABO and Rh blood groups in Nigeria: A review
Egyptian Journal of Medical Human Genetics
2017
18
 3
205
210
10.1016/j.ejmhg.2016.10.004
20

HamedCT
BollahiMA
AbdelhamidI
Med MahmoudMA
BaB


Frequencies and ethnic distribution of ABO and Rh(D) blood groups in Mauritania: results of first nationwide study
International Journal of Immunogenetics
2012
39
 2
151
154
10.1111/j.1744-313X.2011.01064.x
22128837
21

EnawgawB
AynalemM
MelkuM

Distribution of ABO and Rh-D Blood Group Antigens Among Blood Donors in the Amhara Regional State, Ethiopia
Journal of Blood Med
2022
13
97
104
10.2147/JBM.S356425
35237083
PMC888471122

DokuGN
AgbozoWK
AnnorRA
MawudzroPE
AgbeliEE

Frequencies and ethnic distribution of ABO and RhD blood groups in the Volta region of Ghana, towards effective blood bank services
African Health Science
2022
22
 1
641
647
10.4314/ahs.v22i1.74
PMC938251636032446

## Figures and Tables

**Figure:**
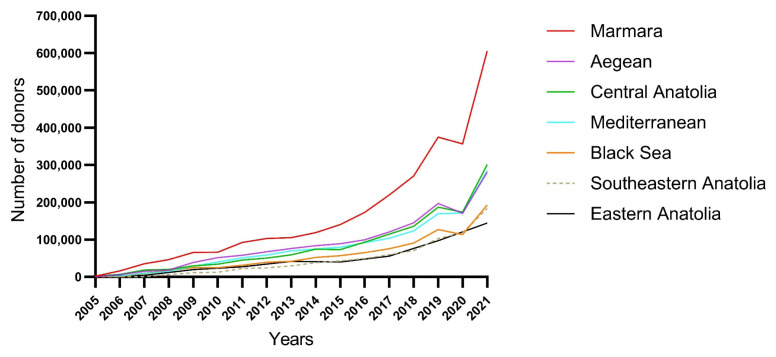
Number of donors by year in different regions.

**Table 1:** Regional distribution of the TRC blood donor demographic data between 1998 and 2021.

Geographic Regions	Male, n (%)	Female, n (%)	Age 18–34 years, n (%)	Age ≥35 years, n (%)	Total, n (%)
**Marmara Region**	2,287,820 (82.0)	503,585 (18.0)	1,289,213 (46.2)	1,502,192 (53.8)	2,791,405 (29.1)
**Aegean Region**	1,227,145 (80.8)	291,681 (19.2)	761,362 (50.1)	757,464 (49.9)	1,518,826 (15.8)
**Central Anatolia Region**	1,141,761 (80.6)	275,662 (19.4)	669,957 (47.3)	747,466 (52.7)	1,417,423 (14.8)
**Mediterranean Region**	1,160,029 (84.3)	215,656 (15.7)	678,541 (49.3)	697,144 (50.7)	1,375,685 (14.3)
**Black Sea Region**	762,035 (78.9)	203,251 (21.1)	531,318 (49.3)	433,968 (50.7)	965,286 (10.1)
**Southeastern Anatolia Region**	657,929 (85.0)	115,651 (15.0)	431,124 (55.7)	342,456 (44.3)	773,580 (8.1)
**Eastern Anatolia Region**	629,249 (84.4)	116,138 (15.6)	478,532 (64.2)	266,855 (35.8)	745,387 (7.8)
**Total**	7,865,968 (82.0)	1,721,624 (18.0)	4,840,047 (50.5)	4,747,545 (49.5)	9,587,592 (100)

**Table 2:** Donation rates of Turkish donors aged 18–70 (TRC and TURKSTAT database 2021).

Geographic Regions	Male, n (%)*	Male, n (%)**	Rate (%)***	Female, n (%)#	Female, n (%)##	Rate (%)###	Age 18–34 years, n (%)§	Age 18–34 years, n (%)§§	Rate (%)§§§	Age ≥35 years, n (%)¥	Age ≥35 years, n (%)¥¥	Rate (%)¥¥¥	Total, n (%)⌂	Total, n (%)⌂⌂	Rate (%)⌂⌂⌂
**Marmara Region**	9,339,890 (50.4)	535,208 (84.7)	5.7	9,193,536 (49.6)	96,906 (15.3)	1.1	6,995,643 (37.7)	305,015 (48.3)	4.4	11,537,783 (62.3)	327,099 (51.7)	2.8	1,853,3426 (32.3)	632,114 (29.5)	3.4
**Aegean Region**	3,805,846 (50.1)	264,842 (86.9)	7.0	3,784,459 (49.9)	40,028 (13.1)	1.1	2,567,085 (33.8)	145,249 (47.6)	5.7	5,023,220 (66.2)	159,621 (52.4)	3.2	7,590,305 (13.2)	304,870 (14.2)	4.0
**Central Anatolia Region**	4,623,619 (49.8)	270,481 (82.3)	5.8	4,652,911 (50.2)	58,177 (17.7)	1.3	3,500,015 (37.7)	162,875 (49.6)	4.7	5,776,515 (62.3)	165,783 (50.4)	2.9	9,276,530 (16.2)	328,658 (15.3)	3.5
**Mediterranean Region**	3,644,616 (50.2)	279,668 (89.7)	7.7	3,616,798 (49.8)	31,977 (10.3)	0.9	2,662,232 (36.7)	149,195 (47.9)	5.6	4,599,182 (63.3)	162,450 (52.1)	3.5	7,261,414 (12.7)	311,645 (14.5)	4.3
**Black Sea Region**	2,777,385 (50.2)	178,495 (84.9)	6.4	2,753,349 (49.8)	31,712 (15.1)	1.2	1,903,078 (34.4)	112,316 (53.4)	5.9	3,627,656 (65.6)	97,891 (46.6)	2.7	5,530,734 (9.6)	210,207 (9.8)	3.8
**Southeastern Anatolia Region**	2,675,452 (50.5)	176,940 (88.1)	5.8	2,610,648 (49.5)	23,996 (11.9)	0.8	2,633,280 (43.6)	116,850 (58.2)	4.4	2,652,820 (56.4)	84,086 (41.8)	2.5	6,046,425 (10.5)	200,936 (9.4)	3.3
**Eastern Anatolia Region**	1,939,923 (51.0)	137,270 (87.1)	8.7	1,847,427 (49.0)	20,384 (12.9)	1.3	1,766,305 (45.6)	101,457 (64.4)	7.2	2,021,045 (54.4)	56,197 (35.6)	3.3	3,096,985 (5.4)	157,654 (7.3)	5.1
**Total**	**28,806,731 (50.3)**	**1,842,904 (85.9)**	**6.4**	**28,459,128 (49.7)**	**303,180 (14.1)**	**1.1**	**22,027,638 (37.8)**	**1,092,957 (50.9)**	**5.0**	**35,238,221 (62.2)**	**1,053,127 (49.1)**	**3.0**	**57,265,859 (100)**	**2,146,084 (100)**	**3.7**

* Total male population aged between 18 and 70 years in Türkiye (TURKSTAT 2021 data)

** The number of males who donated blood to the Turkish Red Crescent in 2021

*** Blood donation rate to the Turkish Red Crescent of Turkish male donors aged between 18 and 70 years in 2021

# Total female population aged between 18 and 70 years in Türkiye (TURKSTAT 2021 data)

## The number of females who donated blood to the Turkish Red Crescent in 2021

### Blood donation rate to the Turkish Red Crescent of Turkish female donors aged between 18 and 70 years in 2021

§ Total population aged between 18 and 34 years in Türkiye (TURKSTAT 2021 data)

§§ The number of donors aged between 18 and 34 years who donated blood to the Turkish Red Crescent in 2021

§§§ Blood donation rate to the Turkish Red Crescent of Turkish donors aged between 18 and 34 years in 2021

¥ Total population aged ≥35 years in Türkiye (TURKSTAT 2021 data)

¥¥ The number of donors aged ≥35 years who donated blood to the Turkish Red Crescent in 2021

¥¥¥ Blood donation rate to the Turkish Red Crescent of Turkish people aged ≥35 years in 2021

⌂ Total population aged between 18 and 70 years in Türkiye (TURKSTAT 2021 data)

⌂⌂ The number of donors who donated blood to the Turkish Red Crescent in 2021

⌂⌂⌂ Blood donation rate to the Turkish Red Crescent of Turkish people in 2021

TURKSTAT database 2021: Address-Based Population Registration System Results, 2021 [online]. Website https://data.tuik.gov.tr/Bulten/Index?p=Adrese-Dayali-Nufus-Kayit-Sistemi-Sonuclari-2021-45500 (In Turkish) [accessed 10 July 2025]

**Table 3:** Educational status of the TRC blood donors between 1998 and 2021 by region.

Education level	Marmara Region, n (%)	Aegean Region, n (%)	Central Anatolia Region, n (%)	Mediterranean Region, n (%)	Black Sea Region, n (%)	Southeastern Anatolia Region, n (%)	Eastern Anatolia Region, n (%)	Total, n (%)
**Literate**	29,586 (1.1)	9515 (0.6)	6685 (0.5)	12,338 (0.9)	6536 (0.7)	17,169 (2.3)	8815 (1.2)	90,644 (1.0)
**Primary school**	505,004 (18.4)	304,731 (20.6)	235,945 (17.6)	263,717 (19.8)	142,327 (15.1)	169,931 (22.5)	89,536 (12.4)	1,711,191 (18.4)
**Middle school**	377,137 (13.7)	210,340 (14.2)	151,241 (11.3)	177,950 (13.4)	106,178 (11.3)	116,660 (15.4)	108,830 (15.1)	1,248,336 (13.4)
**High school**	853,151 (31.0)	444,615 (30.0)	423,486 (31.7)	440,175 (33.1)	282,760 (30.1)	234,899 (31.1)	229,492 (31.9)	2,908,578 (31.2)
**Associate’s degree**	283,354 (10.3)	143,301 (9.7)	108,556 (8.1)	109,660 (8.2)	120,928 (12.9)	63,515 (8.4)	76,793 (10.7)	906,107 (9.7)
**Bachelor’s degree**	612,732 (22.3)	336,923 (22.7)	362,578 (27.1)	295,138 (22.2)	260,358 (27.7)	139,825 (18.5)	191,255 (26.6)	2,198,809 (23.6)
**Master’s degree**	77,778 (2.8)	28,309 (1.9)	34,301 (2.6)	23,031 (1.7)	18,232 (1.9)	12,796 (1.7)	13,592 (1.9)	208,039 (2.2)
**Doctorate**	10,988 (0.4)	4248 (0.3)	15,139 (1.1)	7584 (0.6)	2274 (0.2)	1545 (0.2)	1957 (0.3)	43,735 (0.5)
**Total**	**2,749.730 (100)**	**1,481.982 (100)**	**1,337.931 (100)**	**1,329.593 (100)**	**939,593 (100)**	**756,340 (100)**	**720,270 (100)**	**9,315,439 (100)**

**Table 4:** Blood group distribution of the TRC blood donors between 1998 and 2021 by region.

Blood group	Marmara Region, n (%)	Aegean Region, n (%)	Central Anatolia Region, n (%)	Mediterranean Region, n (%)	Black Sea Region, n (%)	Southeastern Anatolia Region, n (%)	Eastern Anatolia Region, n (%)	Total, n (%)
**A Rh (+)**	1,049,527 (37.6)	572,722 (37.7)	536,118 (37.8)	496,047 (36.1)	366,352 (38.0)	261,997 (33.9)	279,864 (37.5)	3,562.627 (37,1)
**A Rh (−)**	152,649 (5.5)	70,501 (4.6)	71,714 (5.1)	58,841 (4.3)	53,675 (5.6)	31,138 (4.0)	38,308 (5.1)	476,826 (5.0)
**B Rh (+)**	377,847 (13.5)	227,224 (15.0)	205,524 (14.5)	212,746 (15.5)	119,769 (12.4)	134,099 (17.3)	106,539 (14.3)	1,383,748 (14.4)
**B Rh (−)**	55,954 (2.0)	28,271 (1.9)	27,744 (2.0)	25,335 (1.8)	17,001 (1.8)	15,989 (2.1)	14,304 (1.9)	184,598 (1.9)
**AB Rh (+)**	181,389 (6.5)	105,784 (7.0)	97,968 (6.9)	90,861 (6.6)	56,240 (5.8)	52,593 (6.8)	49,611 (6.7)	634,446 (6.6)
**AB Rh (−)**	27,886 (1.0)	13,348 (0.9)	13,802 (1.0)	11,212 (0.8)	8,212 (0.9)	6,440 (0.8)	6,785 (0.9)	87,685 (0.9)
**O Rh (+)**	815,757 (29.2)	443,367 (29.2)	407,436 (28.7)	427,391 (31.1)	296,713 (30.7)	241,303 (31.2)	219,835 (29.5)	2,851,802 (29.7)
**O Rh (−)**	130,396 (4.7)	57,609 (3.8)	57,117 (4.0)	53,252 (3.9)	47,324 (4.9)	30,021 (3.9)	30,141 (4.9)	405,860 (4.2)
**Total**	**2,791,405 (100)**	**1,518,826 (100)**	**1,417,423 (100)**	**1,375,685 (100)**	**965,286 (100)**	**773,580 (100)**	**745,387 (100)**	**9,587,592 (100)**

## Footnotes

1 International Standard Classification of Education [online]. Website https://uis.unesco.org/en/topic/international-standard-classification-education-isced [accessed 10 July 2025]

2 Canadian Blood Services (2023). Surveillance Report [online]. Website https://professionaleducation.blood.ca/en/transfusion/publications/surveillance-report [accessed 10 July 2025].

3 Irish Blood Transfusion Service (2023). Annual Report [online]. Website https://www.giveblood.ie/media/publications/annual_reports/ [accessed 10 July 2025].

4 Hong Kong Red Cross (2022). Annual Report [online]. Website https://www.redcross.org.hk/en/publications/annual_reports_2022pdf.html [accessed 10 July 2025].

5 America’s Blood Centers (2024). U.S. Blood Donation Statistics and Public Messaging Guide [online]. Website https://americasblood.org/statistics_guide/ [accessed 10 July 2025].

6 World Health Organization (2025). Blood safety and availability [online]. Website https://www.who.int/news-room/fact-sheets/detail/blood-safety-and-availability [accessed 10 July 2025].

7 U.S.A. National Heart, Lung, and Blood Institute (2024). Blood Donation: Motivators and Barriers [online]. Website https://www.nhlbi.nih.gov/sites/default/files/publications/blood_donation_motivators_and_barriers.pdf [accessed 10 July 2025].

## References

[1] Babacan M Distribution of ABO Rh Blood Groups In Türkiye and Their Distribution according to Geographical Regions (In Turkish) The Eurasian Journal of Medicine 1968 1 27 36

[2] Sanli K Changes in Blood Groups of Patients Cared for in a Third Level Hospital in Istanbul Over the Years Clin Lab 2025 71 3 10.7754/Clin.Lab.2024.241030 40066545

[3] Ergün A Yardımcı S Distribution of ABO Blood Groups and Rh Factor in Türkiye (In Turkish) Ankara Tıp Mecmuası 1993 46 527 533

[4] Tapan YU Investigation of the distribution of abo and kell blood groups and rh subgroups of patients and donors applied to Dokuz Eylul University Medical Faculty Hospital (In Turkish) Dokuz Eylul University Faculty of Medicine İzmir 2019

[5] Balcı YI Övet G Çövüt İE Goncu F Yilmaz M ABO and Rh Blood Groups Frequency in Denizli Province International Journal of Hematology and Oncology 2010 20 103 105

[6] Altuner Torun Y Kaynar LG Karakukcu C Yay M Kurnaz F ABO and Rh Blood Group Distribution in Kayseri Province, Turkey Turk J Haematol 2012 29 1 97 98 10.5505/tjh.2012.26918 24744637 PMC3986782

[7] Aktaş A Ünlü G Evaluation of the Distribution of ABO and Rh Blood Groups in Sivas Province Cumhuriyet Medical Journal 2021 43 1 55 61 10.7197/cmj.887707

[8] Kader Ç Yolcu S Doğan B Pınarbaşlı M İlanbey B ABO and Rh Blood Groups Distribution in Yozgat City, Turkey Journal of Clinical and Experimental Investigations 2014 5 2 169 172 10.5799/ahinjs.01.2014.02.0384

[9] Gündem NS Ataş E Distribution of ABO and Rh Blood Groups among Patients Admitted to a Gynaecology, Obstetrics and Children Hospital in Konya, Turkey Journal of Clinical & Diagnostic Research 2019 13 3 1 4

[10] Coşkun Y The Distribution of “ABO” and “Rh” Blood Group in Gaziantep Region (In Turkish) Gaziantep Üniversitesı Tıp Fakültesi Dergisi 1990 1 13 15

[11] Arac E Solmaz I Samanci S ABO and Rh blood groups frequency in men, women and neonates in Diyarbakir province Annals of Medical Research 2019 26 12 2876 2880 10.5455/annalsmedres.2019.09.538

[12] Zerin M Karakılçık AZ Nazlıgül Y Frequency of ABO and Rh blood groups in Sanliurfa region (In Turkish) Harran Üniversitesi Tıp Fakültesi Dergisi 2004 1 3 15 17

[13] Alpdemir M Alpdemir MF Kocaöz S Ermiş T Atlı A ABO and Rh Blood Group Dıstrıbutıon In Balıkesır Provınce, Turkey (In Turkish) Balıkesir Sağlık Bilimleri Dergisi 2014 3 2 69 73

[14] Kocak AO Omeroglu M Katipoglu B Akbas I Can NO Blood Group Analysis of Patients Applying to Erzurum Ataturk University Hospital Research and Reports of Medical Science 2017 1 1 1 4

[15] Kuku İ Kaya E Erkurt MA Dikilitaş M Yıldız R ABO and Rh Blood Group Distribution in Malatya Region (In Turkish) İnönü Üniversitesi Tıp Fakültesi Dergisi 2004 11 4 213 215

[16] Akın G Dostbil N Research Of The Blood Groups In Turkey (In Turkish) Yuziincil Yil Universitesi Fen Bilimleri Dergisi 2003 8 1 28 36

[17] Özkasap S Dereci S Şahin K Dilek AR Kalyoncuoğlu E Analysis of ABO and Rh blood groups distribution in East Karadeniz region of Turkey (In Turkiye) Dicle Medical Journal 2013 40 1 100 104 10.5798/diclemedj.0921.2013.01.0232

[18] İnci F Karataş F Distribution of ABO and Rhesus Blood Groups in Cancer Patients (In Turkish) Uludağ Üniversitesi Tıp Fakültesi Dergisi 2020 46 3 379 384 10.32708/uutfd.812217

[19] Yıldız ŞM Distribution of ABO and Rh blood group systems in Cukurova region (In Turkish) Cukurova Medical Journal 2016 41 4 658 663

[20] Koçtekin B Investigation of ABO and Rhesus (Rh) blood group distribution in donors applying to Antalya Education and Research Hospital Transfusion Center (In Turkish). (In Turkish) Mersin Üniversitesi Saglık Bilimleri Dergisi 2020 13 3 395 403

